# Effects of a cognitive and physical intervention program in older adults in Panama

**DOI:** 10.1002/alz.086255

**Published:** 2025-01-03

**Authors:** Elianne S Pauli Quiros, Diana C Oviedo, Gabrielle B Britton, Giselle Rangel, Alcibiades E Villarreal, Jemila Juárez, Hjalmar Jones

**Affiliations:** ^1^ Instituto de Investigaciones Científicas y Servicios de Alta Tecnología (INDICASAT AIP), Centro de Neurociencias, Panama Panama; ^2^ Universidad Santa Maria La Antigua, Panama Panama; ^3^ Instituto de Investigaciones Científicas y Servicios de Alta Tecnología (INDICASAT AIP), Centro de Neurociencias, Panamá, Panamá Panama; ^4^ Sistema Nacional de Investigación (SNI), Panamá, Panamá Panama; ^5^ Universidad Santa María La Antigua, Panamá, Panamá Panama; ^6^ Servicio de Neurología, CIDELAS‐CSS, Panamá, Panamá Panama; ^7^ Universidad de Panamá, Panama Panama

## Abstract

**Background:**

Dementia affects nearly 50 million people worldwide and is the leading cause of disability in older adults. According to predictions by the World Health Organization, the global population with dementia will reach 82 million by 2030, and 152 million by 2050. Currently, pharmacological interventions to delay cognitive decline are proving to be insufficient. By contrast, non‐pharmacological approaches have gained attention in recent years because they can improve clinical symptoms, are low risk and could even alleviate the burden on caregivers. The present study aims to evaluate the effect of an intervention program on cognition, subjective well‐being, and physical health in older adults in Panama.

**Method:**

This is a randomized controlled trial study. The sample includes 60 participants aged 60‐80 years, divided into three groups: 1) a control group, which will receive informative talks about health; 2) experimental group 1, which will participate in cognitive and physical interventions, including cognitive training using the CogniFit platform, group cognitive training and weekly walks monitored with a smartwatch; and 3) experimental group 2, that will perform weekly walks only. A pre‐assessment of the participants is currently being conducted, with 42 participants evaluated so far. The assessment includes a sociodemographic interview, clinical scales, a cognitive test battery, and a physical assessment. Subsequently, a three‐month intervention program will be implemented, followed by a reinforcement program to maintain cognitive and physical training habits introduced in the intervention program. Last, a post‐test will be used to evaluate cognitive function 6 months after the beginning of the intervention program.

**Results:**

According to existing literature, it is expected the group receiving multidomain interventions will improve significantly more than groups receiving a single intervention and the control group.

**Conclusion:**

This study is projected to benefit the physical, cognitive, and emotional health of the participants, as well as to provide them with tools for the prevention of cognitive decline. It will also generate a new line of research that will have an impact on the treatment of older adults with cognitive impairment in Panama.

